# The GBD 2021 perspective: COVID-19’s impact on diarrheal mortality and etiological trends, 1990–2021

**DOI:** 10.3389/fcimb.2025.1668444

**Published:** 2025-11-19

**Authors:** Shaowei Sang, Yuanhui Qiu, Chen Liu, Lili Jiang, Yuheng Zhang, Jun Zheng, Peng Chen

**Affiliations:** 1 Clinical Epidemiology Unit, Qilu Hospital of Shandong University, Jinan, Shandong, China; 2 Department of Epidemiology, School of Public Health, Cheeloo College of Medicine, Shandong University, Jinan, Shandong, China; 3 School of Stomatology, Jining Medical University, Jining, Shandong, China; 4 Department of Healthcare-Associated Infection Management, School and Hospital of Stomatology, Cheeloo College of Medicine, Shandong University, Jinan, Shandong, China; 5 Shandong Key Laboratory of Oral Tissue Regeneration & Shandong Engineering Research Center of Dental Materials and Oral Tissue Regeneration & Shandong Provincial Clinical Research Center for Oral Diseases, Jinan, Shandong, China; 6 Department of Oral and Maxillofacial Surgery, School and Hospital of Stomatology, Cheeloo College of Medicine, Shandong University, Jinan, Shandong, China

**Keywords:** diarrheal diseases, global burden, age-standardized mortality rate, etiology, COVID-19

## Abstract

**Introduction:**

Diarrheal diseases remain a leading cause of global mortality. While significant progress has been made in reducing diarrheal deaths, particularly among children under five, the COVID-19 pandemic introduced new dynamics through non-pharmaceutical interventions and healthcare disruptions. Understanding the evolving burden during this period is critical for guiding post-pandemic control strategies.

**Methods:**

Using data from the Global Burden of Disease Study 2021, we analyzed diarrhea-related deaths and age-standardized death rates (ASDRs) across 204 countries from 1990 to 2021. Estimated annual percentage changes (EAPCs) were calculated to assess temporal trends. Analyses were stratified by age, sex, socio-demographic index (SDI), and 13 major diarrheal pathogens.

**Results:**

Globally, diarrheal deaths declined from 1.26 million (95% UI: 0.90 to 1.72) in 2019 to 1.17 million (0.79 to 1.62) in 2021, with accelerated reduction during the pandemic (EAPC -5.10 vs -4.25 pre-pandemic). Adults >70 years emerged as the highest-risk group (100.75 deaths/100,000), surpassing children under five (51.72/100,000). Eastern Sub-Saharan Africa bore the heaviest burden (ASDR 59.99), while high-income North America showed the fastest pre-pandemic increase (EAPC 8.10). Rotavirus remained the leading cause (0.18 million deaths), though norovirus rose to second place (0.12 million). Pathogen distribution varied markedly by SDI, with *Clostridium difficile* dominating in high-SDI regions (ASDR 0.53) and *Cryptosporidium* in low-SDI areas (ASDR 8.69). The Caribbean saw the most rapid pre-pandemic increase in cholera (EAPC 47.21). Notably, East Asia experienced increasing ASDRs for multiple pathogens during the pandemic, contrasting with global declines. SDI showed strong negative correlations with most pathogens (ρ<-0.8), except for *Clostridium difficile* (ρ=0.63).

**Conclusions:**

Despite accelerated mortality reduction during COVID-19, persistent disparities highlight the dual challenge of pediatric and geriatric diarrheal burdens. Targeted strategies must combine expanded vaccination (particularly for rotavirus and emerging norovirus), SDI-adapted WASH interventions, and strengthened surveillance to address the shifting epidemiological landscape and achieve equitable control.

## Introduction

Diarrheal diseases remain a significant global health issue. The Global Burden of Diseases, Injuries, and Risk Factors Study 2021 (GBD 2021) identified diarrheal diseases as the second leading Level 3 cause of global incidence in 2021, with 4.67 billion (95% uncertainty interval [UI]: 4.11 to 5.22) incident cases reported (GBD 2021 Diseases and Injuries Collaborators, 2024). Importantly, the age-standardized disability-adjusted life-year (DALY) rates decreased by 47.0% (95% UI: 39.9 to 52.9) from 2010 to 2021 (GBD 2021 Diseases and Injuries Collaborators, 2024). This reduction reflects the successful implementation of strategies to manage key risk factors for diarrhea and the expansion of interventions aimed at preventing or treating acute diarrhea. The decline in diarrheal mortality was particularly pronounced among children younger than 5 years (GBD 2016 Diarrhoeal Disease Collaborators, 2018; GBD 2017 Diarrhoeal Disease Collaborators, 2020).

However, the inequitable global distribution of resources available and robust infrastructure for managing the burden of diarrhea results in a disproportionately higher mortality rate in low-income countries compared to high-income countries (Mills, 2014; Berendes et al., 2023; Wolf et al., 2023; Young et al., 2023). Several global initiatives, such as the Global Action Plan for Pneumonia and Diarrhea (GAPPD) and the Sustainable Development Goals, have provided guidance on effective interventions to reduce the incidence and mortality associated with severe diarrhea (WHO UNICEF, 2013; WHO UNICEF, 2017; Aliabadi et al., 2019). Nonetheless, accurate estimates of the burden of diarrhea and its aetiologies are crucial for tracking progress in reducing mortality.

The GBD study represents a systematic, comprehensive, and continually evolving research initiative aimed at quantifying the impact of over 200 diseases and 80 risk factors across various locations, age groups, sexes, and years globally (GBD 2019 Diarrhoeal Disease Collaborators, 2020; Murray, 2022; GBD 2021 Diseases and Injuries Collaborators, 2024). In GBD 2017, updates were made to the mortality associated with diarrheal diseases among children younger than 5 years, and the influences of risk factors, interventions, and broader sociodemographic developments on mortality changes were quantified through GBD’s comparative risk assessment framework (GBD 2017 Diarrhoeal Disease Collaborators, 2020). Nevertheless, the COVID-19 pandemic precipitated unprecedented changes in the epidemiological landscape, necessitating a reevaluation of diarrheal disease patterns. Commencing in 2020, the pandemic emerged as a significant global concern, with non-pharmaceutical interventions (NPIs) such as stay-at-home orders, school and community closures significantly altering the epidemiological characteristics of diarrheal diseases (Geng et al., 2021; Xiao et al., 2021; Adu et al., 2024). The incidence of infections caused by major pathogens, including norovirus and rotavirus, decreased in response to these NPIs, although outbreaks occurred in certain regions as these measures were relaxed (England. PH; Eigner et al., 2021; Hotham, 2021; Nachamkin et al., 2021). In this context, a systematic and up-to-date analysis of the burden of diarrhea, disaggregated by etiology, location, age group, sex, and year assumes an even more important function.

The GBD 2021 represents the first comprehensive assessment incorporating COVID-19’s impact on the burden of selected conditions (GBD 2021 Diseases and Injuries Collaborators, 2024). This study elucidates the findings of GBD 2021 concerning diarrhea, providing estimates of diarrheal mortality and pathogen distribution. We aimed to provide the first thorough assessment of the diarrheal burden that incorporates data from the pandemic-era, thereby addressing critical knowledge gaps in pathogen-specific burden distribution. This assessment encompasses a comprehensive set of 13 etiologies, covering mortality across 204 countries and territories from 1990 to 2021, disaggregated by age and sex.

## Materials and methods

### Study data

Annual deaths and age-standardized death rates (ASDRs) for diarrheal diseases from 1990 to 2021, stratified by age, sex and country, were obtained from the Global Health Data Exchange (GHDx) query tool (http://ghdx.healthdata.org/gbd-results-tool). Diarrheal mortality was defined using ICD-9 and ICD-10 codes (ICD-9: 001-001.9, 003-006.9, 007.4-007.8, 008.01-008.02, 008.04, 008.2-009.9, and 787.91; ICD-10: A00-A00.9, A02-A04.1, A04.3, A04.5-A07, A07.2-A07.4, A08-A09.9, and R19.7) as detailed by GBD collaborators (GBD 2016 Diarrhoeal Disease Collaborators., 2018). A total of 204 countries and territories were categorized into 5 regions based on the socio-demographic index (SDI), which reflected the level of health development across a spectrum including low, low-middle, middle, high-middle, and high. Additionally, these countries and territories were grouped into 21 GBD regions according to socioeconomic similarities and geographical proximity (GBD 2021 Diseases and Injuries Collaborators, 2024).

The methods for the estimations of diarrheal disease mortality had been detailed previously (GBD 2016 Diarrhoeal Disease Collaborators, 2018). In brief, mortality was estimated from surveillance data, vital registration systems, and verbal autopsy using the Cause of Death Ensemble Modelling tool, a Bayesian statistical model that incorporated spatial priors from a hierarchical structure to inform the mortality models. The etiologies of diarrheal diseases, including enteric adenovirus, rotavirus, norovirus, *Cryptosporidium*, *shigella*, enterotoxigenic *E. coli*, cholera, *Campylobacter*, enteropathogenic *E. coli*, *Entamoeba*, non-typhoidal *salmonella*, *Aeromonas*, and *Clostridium difficile*, were estimated from overall diarrheal mortality using a counterfactual strategy based on population attributable fractions (PAFs). For each etiology, PAFs were calculated as:


PAF=Proportion ×(1−1/OR)

where Proportion represents the modelled prevalence of pathogen detection among diarrheal cases, estimated using Bayesian meta-regression (DisMod-MR 2.1) models for each age, sex, year, and location, and OR is the odds ratio of diarrhea given pathogen detection, derived from molecular diagnostic data from the Global Enteric Multicenter Study (GEMS). This approach accounts for pathogen co-detection and asymptomatic carriage, avoiding a one-pathogen-per-episode assumption. Pathogen-specific deaths were then obtained by multiplying total diarrheal deaths by the corresponding PAF. Reported case numbers and rates included 95% uncertainty intervals (UIs), derived from the 2.5th and 97.5th percentiles of 1,000 sample estimates.

### Statistical analysis

Trends in ASDRs were assessed using the estimated annual percentage change (EAPC). The logarithmic ASDR was fitted to a regression line, given by *ln(y) = α+βx+ϵ*, where *y* represented ASDR, and *x* represented the calendar year. EAPC was calculated as *100 × (exp (β)-1)*, and its 95% confidence interval (CI) also derived from the model (Hankey et al., 2000). This metric was selected to ensure comparability with previous GBD studies and to provide a single, interpretable summary of the average annual rate of change over a defined period. While joinpoint regression is useful for detecting inflection points in temporal trends, our analysis focused on comparing two predefined time periods: the long-term secular trend (1990-2019) and the acute pandemic phase (2019-2021). The EAPC approach is particularly suitable for such direct epoch comparisons. Notably, the 2019–2021 interval captures a short-term disruption; thus, its EAPC should be interpreted as the average rate of change during this exceptional period rather than an extension of long-term trends. In contrast, the 1990–2019 EAPC reflects a more stable, long-term trajectory. An increasing trend was identified if the 95% CI of the EAPC estimate was greater than 0, a decreasing trend if less than 0, and a stable trend if the CI including 0. Spearman’s test was used to explore the association between ASDR and SDI. All analyses were conducted using the R program (R core team, version 4.0.2, Vienna, Austria).

The GBD 2021 study is a publicly available database and all data were anonymous. Therefore, the study is exempted from institutional ethical board review.

## Results

### Mortality of diarrheal diseases

In 2019, prior to the onset of the COVID-19 pandemic, the number of deaths attributable to diarrheal diseases was 1.26 million (95% UI: 0.90 to 1.72), corresponding to an ASDR of 17.12 (95% UI: 12.41 to 22.96) per 100,000 population. By 2021, these figures had decreased to 1.17 million (95% UI: 0.79 to 1.62) deaths and an ASDR of 15.42 (95% UI: 10.87 to 20.91) per 100,000 population, as detailed in **Table 1**. This period witnessed a notable reduction in ASDR, with an EAPC of -5.10 (95% CI: -6.03 to -4.15) from 2019 to 2021, surpassing the EAPC of -4.25 (95% CI: -4.42 to -4.08) recorded from 1990 to 2019.

**Table 1:** Deaths and age-standardized death rate of diarrheal diseases for all ages in 1990, 2019 and 2021, and its estimated annual percentage change from 1990 to 2019 and from 2019 to 2021, globally, SDI regions and GBD regions.

Location	1990		2019		2021		1990-2019	2019-2021
	Deaths No. (95% UI)	Age-standardized death rate per 100 000 No. (95% UI)	Deaths No. (95% UI)	Age-standardized death rate per 100 000 No. (95% UI)	Deaths No. (95% UI)	Age-standardized death rate per 100 000 No. (95% UI)	EAPC in Age-standardized death rate No. (95% CI)	
Global	2932253 (2308355, 3730011)	60.58 (46.34, 79.9)	1263033 (895348, 1723172)	17.12 (12.41, 22.96)	1165398 (793421, 1618358)	15.42 (10.87, 20.91)	-4.25 (-4.42, -4.08)	-5.1 (-6.03, -4.15)
SDI region								
High SDI	7296 (6416, 8260)	0.84 (0.73, 0.97)	26417 (22247, 28869)	1.18 (1.02, 1.28)	26789 (22379, 29454)	1.13 (0.98, 1.24)	2.75 (1.92, 3.59)	-1.95 (-2.63, -1.26)
High-middle SDI	46989 (36645, 56615)	5.26 (4.09, 6.33)	16696 (12032, 20330)	1.14 (0.86, 1.37)	16557 (11694, 20632)	1.09 (0.82, 1.35)	-5.56 (-5.99, -5.14)	-2.13 (-2.54, -1.72)
Middle SDI	508460 (382882, 645256)	41.96 (28.5, 58.22)	170497 (106079, 235600)	8.28 (5.23, 11.38)	165735 (100175, 237699)	7.69 (4.8, 10.82)	-5.49 (-5.58, -5.41)	-3.6 (-4.96, -2.23)
Low-middle SDI	1476055 (1165970, 1919146)	185.39 (136.59, 261.93)	526766 (358738, 786222)	42.7 (28.06, 65.26)	482680 (317411, 736393)	38.41 (24.75, 59.72)	-4.95 (-5.09, -4.81)	-5.16 (-6.1, -4.22)
Low SDI	892003 (696869, 1120386)	226.43 (166.82, 311.5)	521979 (391861, 682862)	77.82 (51.37, 115.9)	472987 (350646, 628593)	69.75 (44.84, 103.46)	-3.58 (-3.78, -3.37)	-5.33 (-5.51, -5.15)
GBD region								
High-income North America	858 (784, 907)	0.28 (0.26, 0.29)	10011 (8555, 10831)	1.5 (1.3, 1.61)	10255 (8779, 11079)	1.48 (1.29, 1.59)	8.1 (6.38, 9.85)	-0.77 (-1.45, -0.07)
Australasia	140 (127, 153)	0.68 (0.62, 0.75)	291 (243, 323)	0.52 (0.45, 0.58)	296 (244, 330)	0.49 (0.41, 0.54)	1.07 (-0.23, 2.39)	-3.27 (-8.29, 2.03)
High-income Asia Pacific	1618 (1292, 1917)	1.08 (0.87, 1.26)	4587 (3542, 5678)	0.83 (0.67, 1.03)	4823 (3684, 6009)	0.8 (0.64, 1)	-0.18 (-0.48, 0.12)	-1.88 (-4.62, 0.95)
Western Europe	2721 (2439, 2907)	0.52 (0.47, 0.56)	13386 (11154, 14811)	1.18 (1.01, 1.29)	13245 (10914, 14688)	1.11 (0.94, 1.22)	4.15 (3.2, 5.11)	-2.84 (-3.83, -1.85)
Southern Latin America	1599 (1511, 1680)	3.49 (3.29, 3.67)	1368 (1229, 1488)	1.7 (1.54, 1.84)	1264 (1119, 1399)	1.52 (1.35, 1.68)	-1.94 (-2.5, -1.38)	-5.56 (-7.08, -4.01)
Eastern Europe	2040 (1967, 2113)	1.16 (1.12, 1.21)	562 (539, 584)	0.24 (0.24, 0.26)	550 (513, 587)	0.24 (0.23, 0.26)	-7.11 (-7.88, -6.33)	-0.76 (-2.42, 0.93)
Central Europe	1028 (934, 1136)	1.11 (1, 1.23)	2603 (2345, 2833)	1.44 (1.33, 1.55)	2566 (2272, 2834)	1.37 (1.22, 1.51)	0.83 (-0.93, 2.62)	-2.6 (-3.14, -2.05)
Central Asia	14265 (12909, 15884)	15.52 (14.08, 17.26)	2072 (1523, 2741)	2.12 (1.56, 2.8)	1913 (1412, 2561)	1.95 (1.45, 2.6)	-8.01 (-8.43, -7.57)	-4.06 (-7.02, -1.01)
Central Latin America	55961 (52636, 59878)	35.7 (33.87, 37.7)	12698 (11401, 14176)	5.72 (5.12, 6.4)	11896 (10243, 13903)	5.25 (4.48, 6.17)	-6.3 (-6.88, -5.71)	-4.19 (-6.59, -1.73)
Andean Latin America	10064 (8536, 11892)	24.64 (20.18, 29.42)	1888 (1363, 2806)	3.25 (2.34, 4.82)	1681 (1174, 2599)	2.86 (1.99, 4.41)	-7.39 (-7.69, -7.08)	-6.14 (-8.1, -4.13)
Caribbean	14656 (12351, 17176)	37.84 (32.09, 44.4)	5986 (4242, 8129)	14.29 (10.14, 19.68)	5803 (4093, 7851)	13.87 (9.74, 18.73)	-3.06 (-3.55, -2.56)	-1.51 (-1.98, -1.03)
Tropical Latin America	37842 (33327, 42464)	27.72 (25.04, 30.59)	6463 (5771, 6943)	2.97 (2.66, 3.21)	6176 (5411, 6659)	2.68 (2.35, 2.9)	-7.8 (-8.2, -7.39)	-5.09 (-5.14, -5.04)
East Asia	91455 (67839, 113073)	9.01 (6.5, 11.39)	4677 (3049, 8067)	0.34 (0.24, 0.54)	4500 (2880, 7645)	0.34 (0.24, 0.51)	-11.69 (-12.08, -11.29)	-1.34 (-5.5, 3.01)
Southeast Asia	317054 (211742, 461497)	98.38 (57.22, 152.66)	77401 (43685, 102219)	15.26 (8.21, 20.35)	75513 (41686, 100810)	14.22 (7.59, 19.25)	-6.18 (-6.37, -5.99)	-3.48 (-3.82, -3.14)
Oceania	3103 (2170, 4166)	79.04 (48.81, 109.52)	3411 (2290, 4697)	41.79 (25.62, 59.69)	3364 (2234, 4751)	39.37 (23.86, 55.89)	-1.9 (-2.11, -1.68)	-2.93 (-3.57, -2.29)
North Africa and Middle East	97166 (74533, 122743)	21.87 (16.67, 27.16)	19480 (14672, 26442)	3.67 (2.72, 4.88)	15995 (11729, 21848)	3.15 (2.28, 4.3)	-6.34 (-6.54, -6.13)	-7.37 (-10.06, -4.59)
South Asia	1487204 (1163614, 1996412)	230.96 (169.95, 330.02)	616247 (409451, 954421)	53.45 (35.26, 83.55)	571732 (368043, 902337)	47.72 (30.52, 75.95)	-4.95 (-5.11, -4.79)	-5.51 (-6.28, -4.74)
Southern Sub-Saharan Africa	37899 (31314, 46953)	82.24 (60.05, 116.63)	25491 (18086, 34689)	43 (28.54, 61.93)	23945 (16621, 33164)	39.93 (26.13, 57.63)	-1.89 (-2.22, -1.56)	-3.63 (-4.37, -2.89)
Western Sub-Saharan Africa	380657 (271465, 466132)	188.92 (127.39, 258.31)	257368 (183029, 338019)	63.88 (43.71, 87.8)	229576 (162877, 305831)	56.97 (38.37, 80.13)	-3.7 (-4, -3.41)	-5.56 (-6.7, -4.41)
Eastern Sub-Saharan Africa	296381 (212912, 393721)	180.6 (113.91, 263.03)	162585 (117276, 212836)	65.78 (38.57, 89.98)	150239 (105248, 202390)	59.99 (34.56, 81.73)	-3.57 (-3.71, -3.42)	-4.5 (-4.91, -4.09)
Central Sub-Saharan Africa	78545 (56824, 97672)	148.47 (100.81, 202.43)	34456 (22308, 49070)	43.9 (25.33, 68.68)	30067 (18591, 44363)	39.29 (22.37, 61.25)	-3.96 (-4.65, -3.26)	-5.4 (-5.73, -5.07)

No., number; UI, uncertainty interval; EAPC, estimated annual percentage change; CI, confidential interval; SDI, sociodemographic index.

At the SDI region level, the low-middle SDI region consistently reported the highest number of deaths from 1990 to 2021. Conversely, the low SDI region exhibited the highest ASDR (per 100,000 population), with values of 226.43 (95% UI: 166.82 to 311.50) in 1990, 77.82 (95% UI: 51.37 to 115.90) in 2019, and 69.75 (95% UI: 44.84 to 103.46) in 2021. However, between 1990 and 2019, the high-middle SDI region experienced the most substantial decline in ASDR, with an EAPC of -5.56 (95% CI: -5.99 to -5.14), while the high SDI region was the sole region to exhibit a significant increase in ASDR (EAPC 2.75 [95% CI: 1.92 to 3.59]). From 2019 to 2021, all ASDRs decreased among the SDI regions, with the largest decrease observed in the low SDI region (EAPC -5.33 [95% CI: -5.51 to -5.15]). Together, these data underscore that the diarrheal mortality burden is overwhelmingly concentrated in low-SDI regions, highlighting profound global inequity.

Among the 21 GBD regions, Eastern Sub-Saharan Africa, Western Sub-Saharan Africa, and South Asia exhibited the highest ASDR (per 100,000 population) in 2021, with values of 59.99 (95% UI: 34.56 to 81.73), 56.97 (95% UI: 38.37 to 80.13), and 47.72 (95% UI: 30.52 to 75.95), respectively. Conversely, the most pronounced reductions in ASDR from 1990 to 2019 were recorded in East Asia (EAPC -11.69 [95% CI: -12.08 to -11.29]), and in North Africa and the Middle East from 2019 to 2021 (EAPC -7.37 [95% CI: -10.06 to -4.59]) (**Table 1**). It is noteworthy that high-income North America experienced the most significant increase in ASDR from 1990 to 2019, with an EAPC of 8.10 (95% CI: 6.38 to 9.85), although a slight decline was observed from 2019 to 2021 (EAPC -0.77 [95% CI: -1.45 to -0.07]).

**Figure 1:**
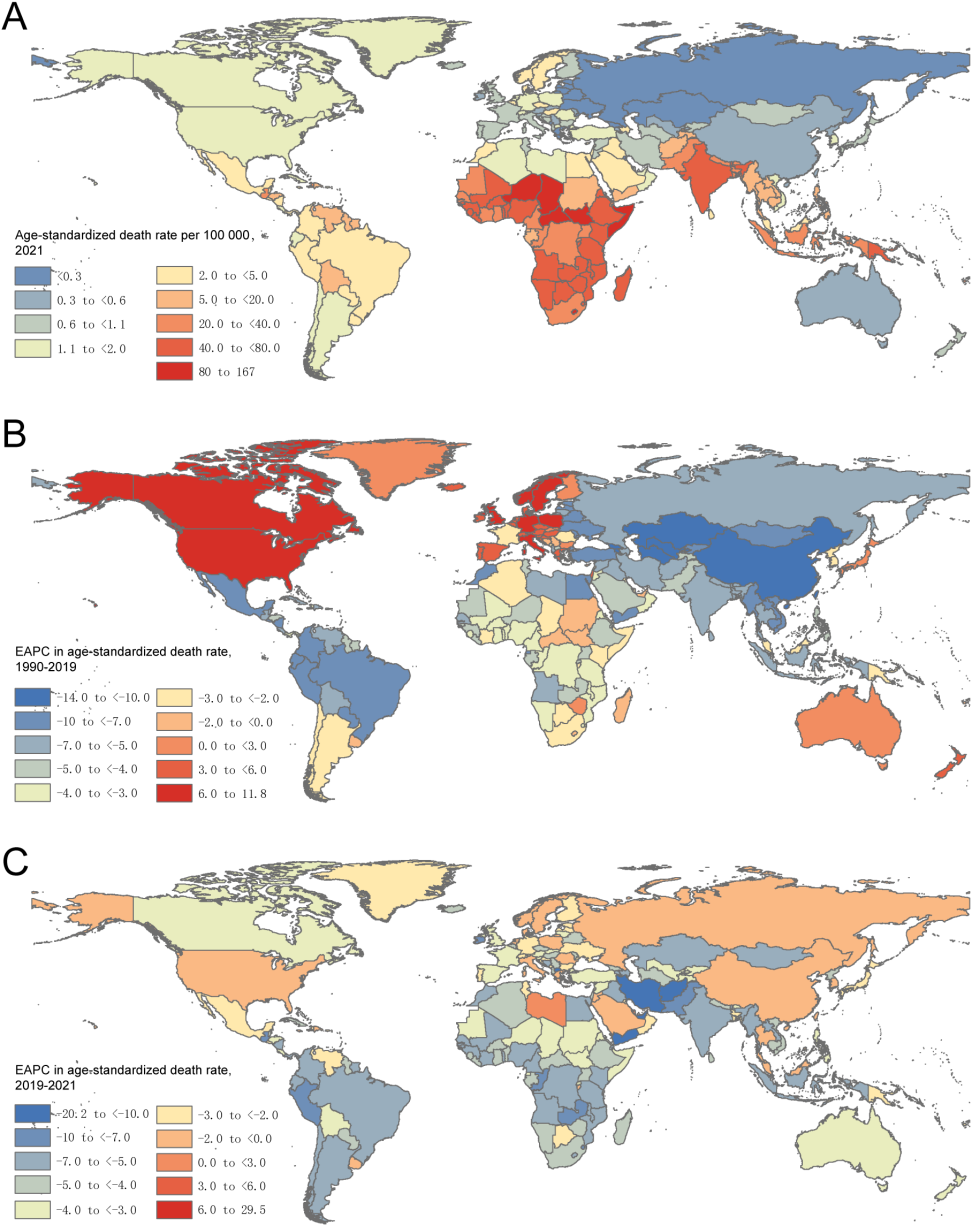
Diarrhoeal diseases age-standardized mortality rate in 2021 **(A)** and its EAPCs in 1990-2019 **(B)** and in 2019-2021 **(C)**, respectively. EAPC, estimated annual percentage change.

At the country or territory level, in 2021, the ASDR per 100,000 population varied from 0.06 (95% UI: 0.04 to 0.10) in Montenegro to 166.68 (95% UI: 98.13 to 277.54) in the Republic of South Sudan (**Figure 1A**). Between 1990 and 2019, the Kingdom of Sweden, the Republic of Italy, and Canada had the most rapid increases in ASDR, with EAPCs of 11.74 (95% CI: 10.48 to 13.02), 10.46 (95% CI: 8.25 to 12.71), and 9.27 (95% CI: 6.99 to 11.62), respectively (**Figure 1B**). From 2019 to 2021, Tokelau and the Republic of Niue ranked the top two, with EAPCs of 29.47 (95% CI: -4.38 to 75.31) and 9.08 (95% CI: -1.82 to 21.19) (**Figure 1C**).

**Figure 2:**
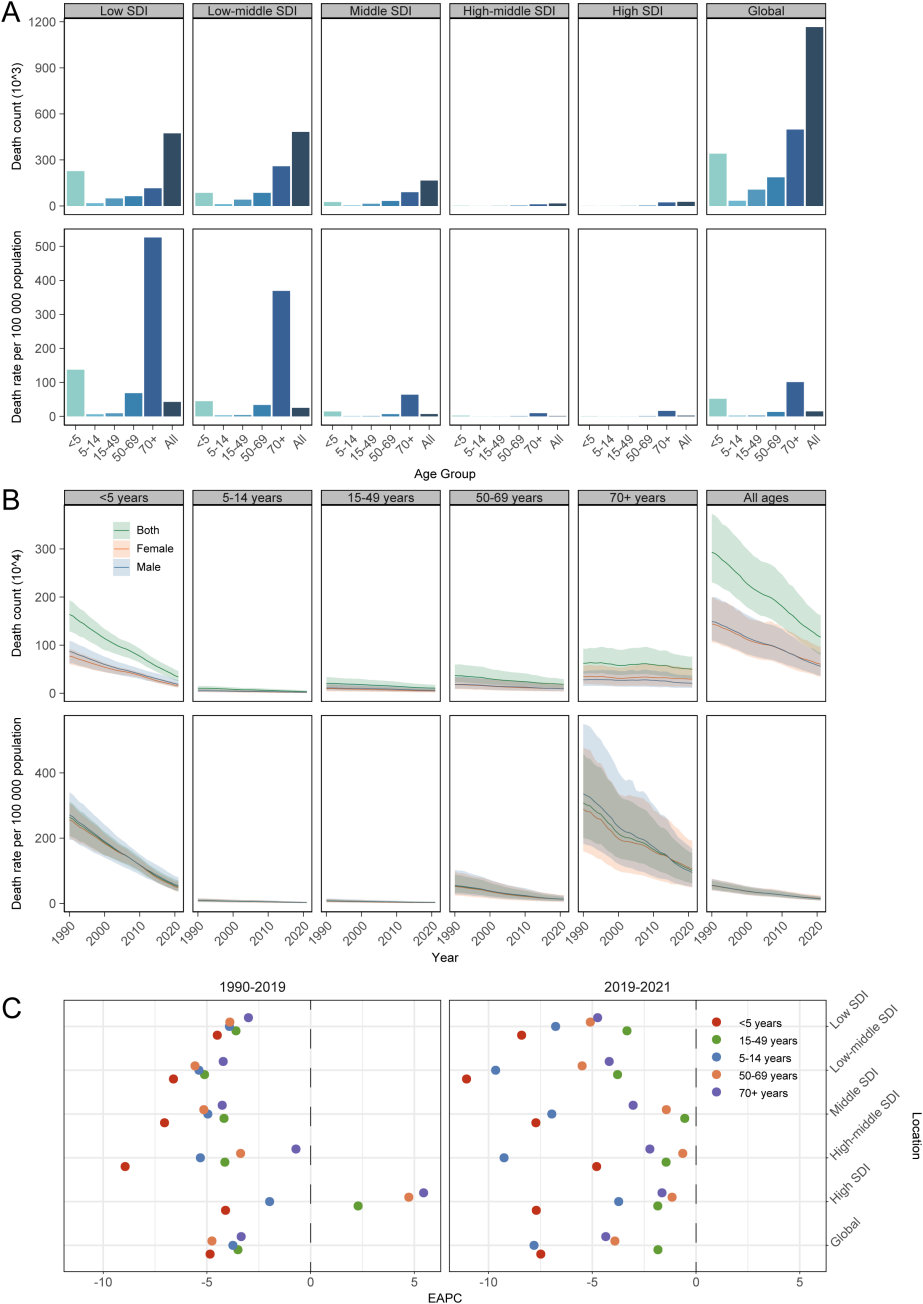
Global age pattern of diarrheal diseases. **(A)**, diarrheal diseases death counts (top) and mortality rates (bottom) by SDI region, 2021; **(B)**, diarrheal diseases death counts (top) and mortality rates (bottom) by sex, 1990-2021; **(C)**, EAPCs in mortality rate in 1990-2019 (left) and 2019-2021 (right) by SDI region.

In 2021, the global mortality data indicated that the highest number and rate of deaths were recorded among adults older than 70 years, with 0.50 million (95% UI: 0.31 to 0.76) deaths and a mortality rate of 100.75 (95% UI: 61.91 to 154.15) per 100,000 population. Similarly, significant mortality was observed among children younger than 5 years, with 0.34 million (95% UI: 0.25 to 0.46) deaths and a mortality rate of 51.72 (95% UI: 38.13 to 70.54) per 100,000 population. This trend was also evident across the SDI regions in 2021. The low-middle SDI region reported the highest number of deaths among adults older than 70 years, totaling 0.26 million (95% UI: 0.16 to 0.42), whereas the low SDI region had the highest mortality rate of 525.90 (95% UI: 311.47 to 864.28) per 100,000 population among the same age group (**Figure 2A**). Collectively, these data showed that adults aged ≥70 years have now surpassed children under five as the highest-risk group for diarrheal mortality.

Globally, between 1990 and 2021, there was a decline in diarrheal deaths for both males and females. Specifically, male deaths decreased from 1.49 million (95% UI: 1.10 to 1.99) to 0.56 million (95% UI: 0.37 to 0.84), while female deaths declined from 1.44 million (95% UI: 1.06 to 2.00) to 0.60 million (95% UI: 0.35 to 0.97). This trend was predominantly driven by a reduction in deaths among children younger than 5 years (**Figure 2B**). The most significant decrease in death rates was observed in the high-middle SDI region from 1990 to 2019 (EAPC -8.94 [95% CI: -9.13 to -8.76]), and in the low-middle SDI region from 2019 to 2021 (EAPC -11.06 [95% CI: -15.62 to -6.26]). Notably, the increase in ASDR in the high SDI region was attributed to rising mortality rates among the population aged 15 years and older from 1990 to 2019. In contrast, the decline in mortality between 2019 and 2021 was primarily due to reduced death rates among children younger than 5 years and those aged 5–14 years, as these age groups exhibited the highest EAPCs across the SDI regions (**Figure 2C**).

### Etiologies of diarrheal diseases

**Figure 3:**
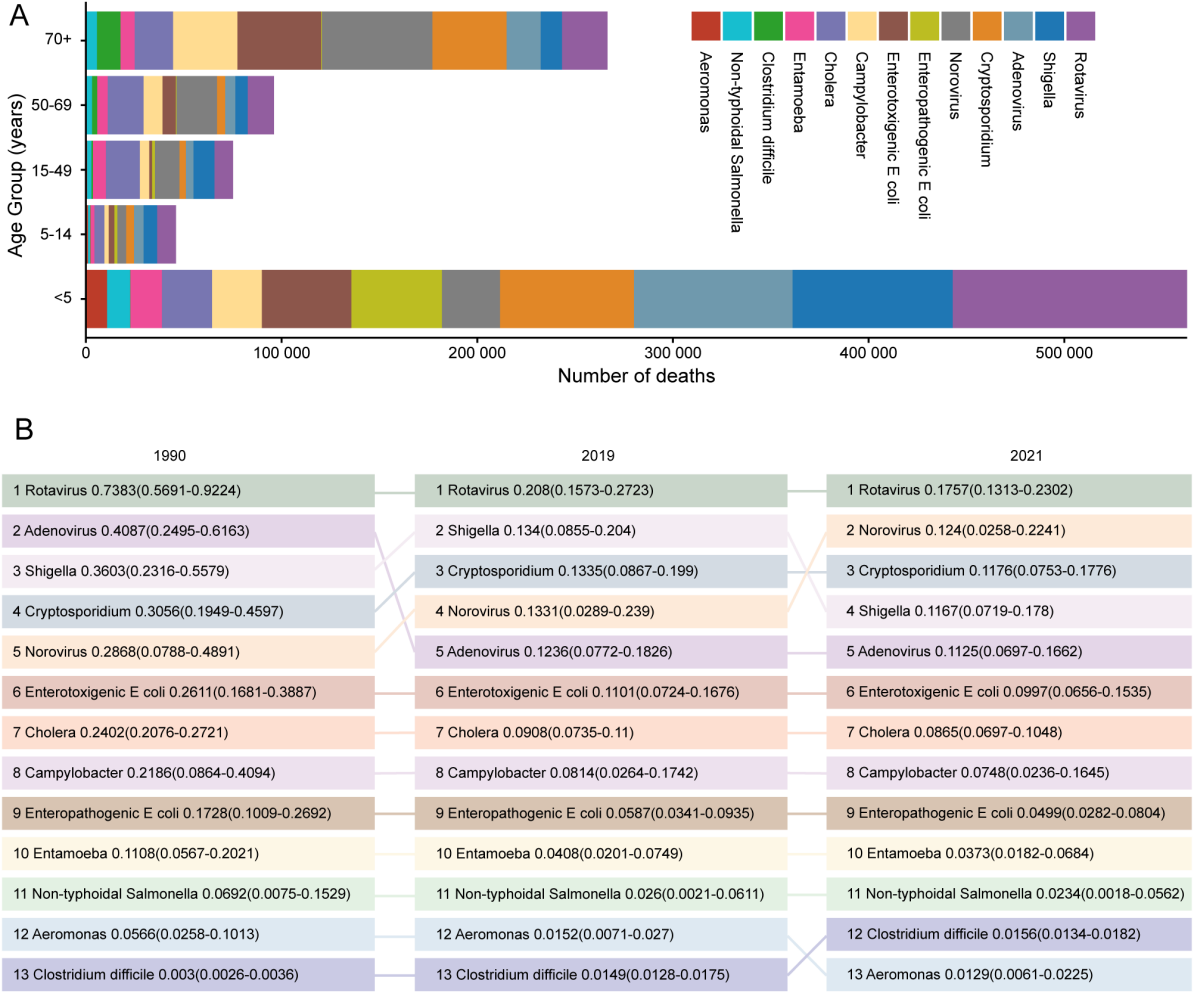
Etiology distribution of global diarrheal diseases deaths by age group in 2021 **(A)** and ranked aetiologies by number of global diarrheal diseases deaths across all ages, 1990, 2019, 2021 **(B)**. Values are estimated millions of deaths caused by each pathogen, with 95% uncertainty intervals in parentheses.

In 2021, the pathogen responsible for the largest proportion of diarrheal deaths globally was rotavirus, which caused an estimated 0.18 million (95% UI: 0.13 to 0.23) deaths. This was followed by norovirus (0.12 million [95% UI: 0.03 to 0.22] deaths) and *Cryptosporidium* (0.12 million [95% UI: 0.08 to 0.18] deaths). Key pathogens varied by age. Rotavirus and norovirus were responsible for the largest number of deaths in children younger than 14 years and in the population aged 50 years and older, respectively, whereas cholera caused the most deaths in the population aged 15–49 years (**Figure 3A**).

During the COVID-19 pandemic in 2021, the top five most common etiology categories were the same as those for 2019 and 1990. Of these, rotavirus was consistently ranked first across all age groups, although deaths attributable to this pathogen decreased from 0.74 million (95% UI: 0.57 to 0.92) in 1990 to 0.18 million (95% UI: 0.13 to 0.23) in 2021 (**Figure 3B**). For norovirus, the deaths burden relative to other etiologies is likely increasing in recent years. The pathogen ranked as the fifth most common etiology of deaths in 1990 (0.29 million [95% UI: 0.08 to 0.49]), while its ranking increased to fourth in 2019 (0.13 million [95% UI: 0.03 to 0.24]) and second in 2021 (0.12 million [95% UI: 0.03 to 0.22]). However, the ranking of adenovirus, decreased from second in 1990 (0.41 million [95% UI: 0.25 to 0.62]) to fifth in 2019 (0.12 million [95% UI: 0.08 to 0.18]) and remained fifth in 2021 (0.11 million [95% UI: 0.07 to 0.17]) (**Figure 3B**). Collectively, rotavirus remained the leading cause of diarrheal death, while norovirus rose to become the second most common etiology.

**Figure 4:**
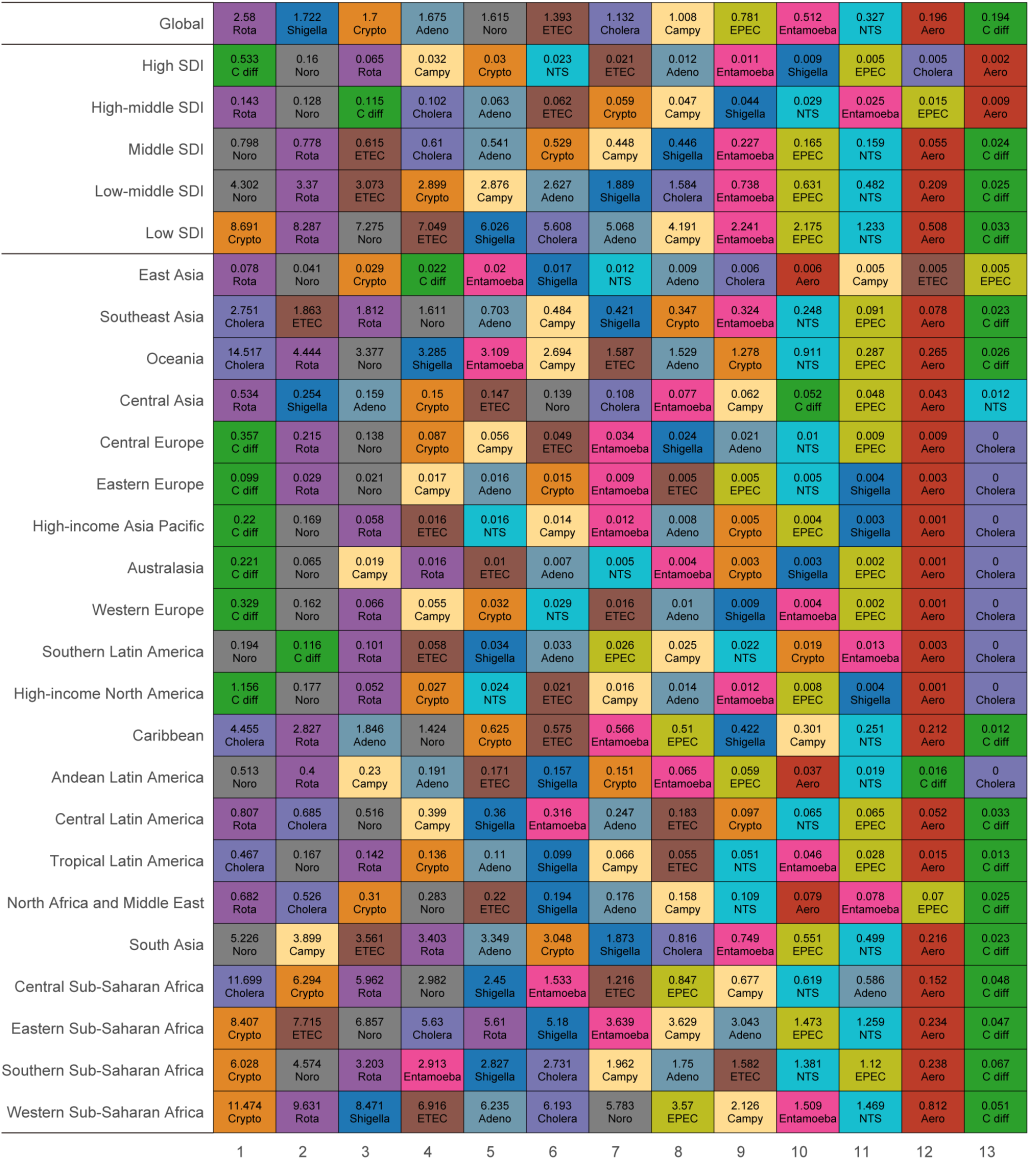
Ranked aetiologies by age-standardized mortality rate of diarrheal diseases across all ages by global, SDI region, GBD region. NTS, Non-typhoidal Salmonella; Adeno, Adenovirus; Aero, Aeromonas; Campy, Campylobacter; C diff, Clostridium difficile; Crypto, Cryptosporidium; EPEC, enteropathogenic E. coli; ETEC, enterotoxigenic E. coli; Noro, Norovirus; Rota, Rotavirus.

Globally in 2021, rotavirus was responsible for the highest ASDR from diarrheal diseases across all ages, with an ASDR of 2.58 (95% UI: 1.92 to 3.42), followed by *Shigella* (1.72 [95% UI: 1.06 to 2.62]), *Cryptosporidium* (1.70 [95% UI: 1.10 to 2.53]), adenovirus (1.68 [95% UI: 1.03 to 2.52]), and norovirus (1.62 [95% UI: 0.35 to 2.91]) (**Figure 4**). The burden of different diarrheal disease aetiologies varied across the SDI regions. *Clostridium difficile* ranked first in the high SDI region (ASDR 0.53 [95% UI: 0.47 to 0.61]) and third in the high-middle SDI region (ASDR 0.12 [95% UI: 0.09 to 0.14]), but ranked last in the other three SDI regions. Norovirus and rotavirus consistently ranked among the top three etiologies across all SDI regions. Most strikingly, *Cryptosporidium* ranked first in the low SDI region, with an ASDR of 8.69 (95% UI: 5.23 to 13.67) (**Figure 4**). At the GBD region level, the major diarrheal death burden attributed to the thirteen pathogens in 2021 was significantly concentrated in Sub-Saharan Africa and Oceania, with ASDRs exceeding 6.00 per 100,000 population. Among these regions, *Cryptosporidium* was the leading cause of ASDR in Sub-Saharan Africa, while in Oceania and Central Sub-Saharan Africa, cholera ranked first, with ASDRs of 14.52 (95% UI: 10.38 to 18.09) and 11.70 (95% UI: 7.92 to 15.81) (**Figure 4**). Additionally, cholera caused a significant burden in the Caribbean and Southeast Asia, with ASDRs of 4.46 (95% UI: 3.29 to 5.78) and 2.75 (95% UI: 2.11 to 3.35) per 100,000 population, respectively. At the country or territory level, the Federal Republic of Somalia recorded the highest ASDR for cholera at 41.45 (95% UI: 32.64 to 51.92) per 100,000 population, followed by the Central African Republic at 34.68 (95% UI: 25.14 to 45.02) per 100,000 population. *Cryptosporidium* was notably prevalent in countries like the Republic of the Niger (ASDR 27.84 [95% UI: 15.31 to 46.04] per 100,000 population) and the Republic of South Sudan (24.66 [95% UI: 13.72 to 43.33] per 100,000 population). In contrast, rotavirus and enterotoxigenic *E. coli* were more commonly reported in the Republic of Chad and Republic of the Niger (ASDR 26.48 [95% UI: 17.01 to 41.40] and 25.81 [95% UI: 14.72 to 41.86] per 100,000, respectively) (**Supplementary Figure S1**).

**Figure 5:**
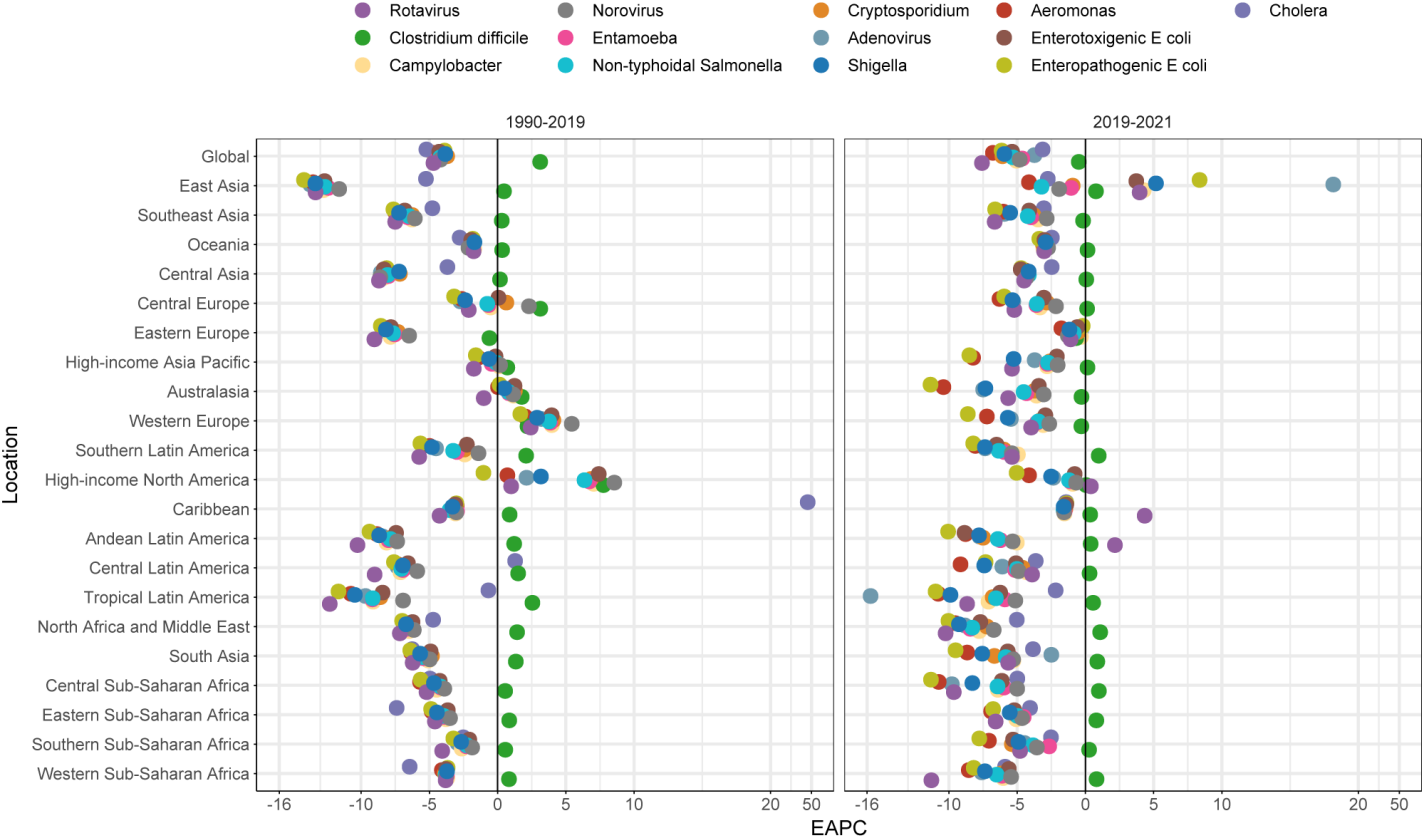
EAPCs in age-standardized mortality rate in 1990-2019 (left) and 2019-2021 (right) by global, GBD region.

Before the COVID-19 pandemic, the ASDRs of the pathogens studied decreased globally, except for *Clostridium difficile*, for which the EAPC was 3.12 (95% CI: 2.54 to 3.70) from 1990 to 2019 (**Figure 5**). Specifically, this increase was observed across all GBD regions, except for Eastern Europe, which experienced a slight decline (EAPC -0.60 [95% CI: -1.08 to -0.12]). In Western Europe, High-income North America, and Australasia, almost all the studied pathogens showed an increasing trend in ASDR from 1990 to 2019. Notably, in the Caribbean, cholera exhibited the fastest rise in ASDR, with an EAPC of 47.21 (95% CI: 37.44 to 57.68). During the COVID-19 pandemic, the ASDRs of the pathogens decreased globally, with EAPC ranging from -7.58 (95% CI: -9.64 to -5.48) for rotavirus to -0.49 (95% CI: -1.14 to 0.17) for *Clostridium difficile* (**Figure 5**). This period saw significant decreases in the ASDR of almost all pathogens, including *Clostridium difficile*, across GBD regions compared to the period before COVID-19. However, during the pandemic, the ASDR of adenovirus, enteropathogenic *E. coli*, *Shigella*, *Campylobacter*, rotavirus, and enterotoxigenic *E. coli* increased in East Asia, with EAPC ranging from 3.73 to 18.16 (**Figure 5**). At the country or territory level, from 1990 to 2019, the fastest declines in enteric pathogens were predominantly observed in Armenia, Kazakhstan, and Uzbekistan, particularly for pathogens such as rotavirus, enteropathogenic *E. coli*, and adenovirus. In contrast, Sweden and Italy showed some of the fastest increases in ASDR for multiple pathogens. From 2019 to 2021, both the Islamic Republic of Iran and Republic of the Congo showed the most substantial decreases in ASDR for enteropathogenic *E. coli*, with EAPCs of -31.45 (95% CI: -32.48 to -30.40) and -23.87 (95% CI: -39.27 to -4.55), respectively (**Supplementary Figure S2**).

**Figure 6:**
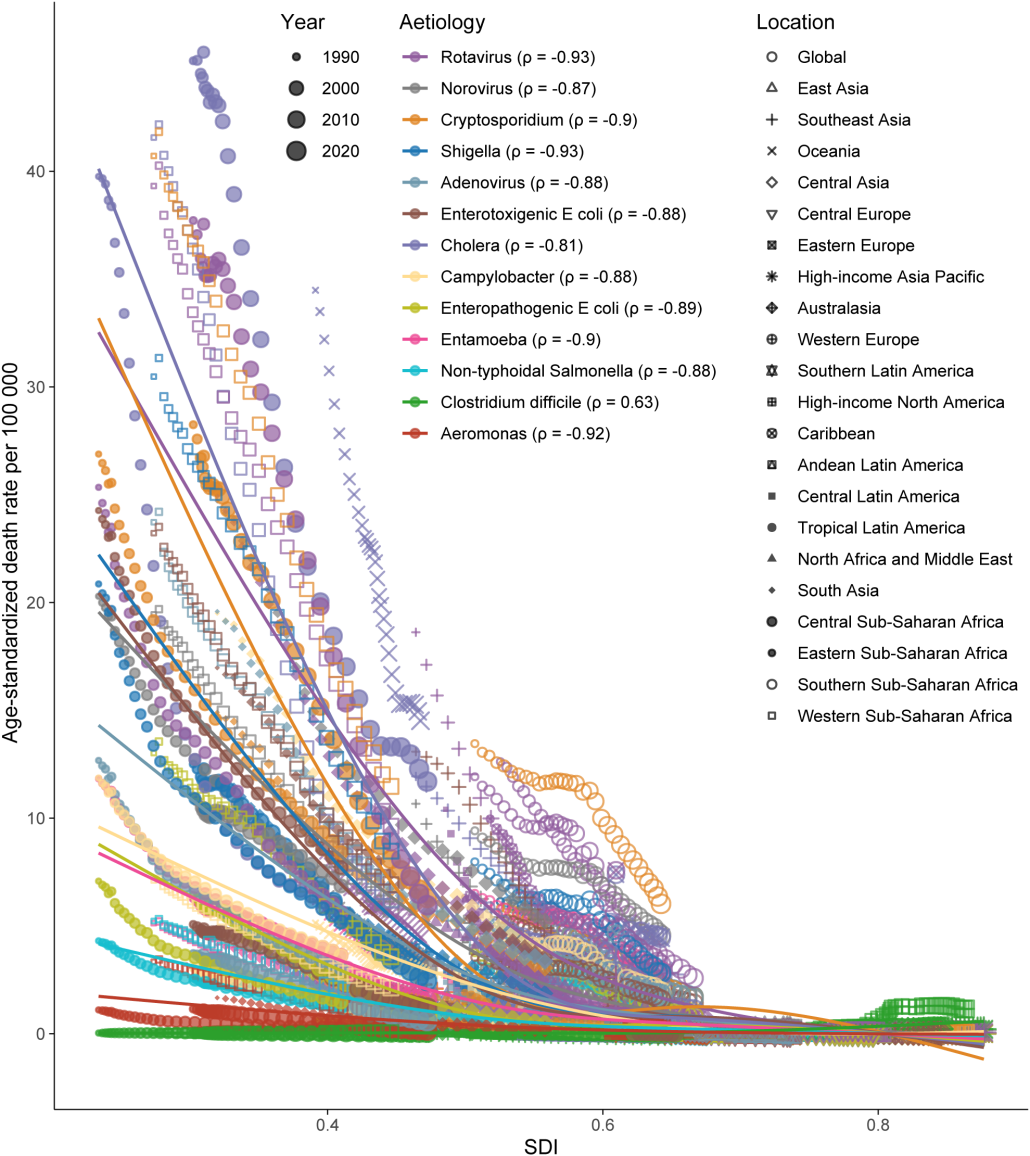
Association between the SDI and diarrheal diseases age-standardized mortality rate across all ages by pathogen (spearman coefficients in parentheses) and region, 1990-2021. SDI, Socio-demographic Index. GBD, Global Burden of Disease. The colored lines are estimated by a least squares cubic spline regression.

Additionally, we analyzed the correlation between SDI and ASDR across 21 GBD regions by etiology, and found significant negative correlations (*ρ* < -0.8), except for *Clostridium difficile*, for which the ASDR was positively correlated with SDI (*ρ* = 0.63) (**Figure 6**). This positive correlation largely contributed to a strongly positive correlation in Western Europe (**Supplementary Figure S3**).

## Discussion

Despite notable advancements in sanitation and public health awareness, diarrhea continues to be a leading cause of morbidity and mortality globally. This study offers a comprehensive analysis of diarrheal deaths and mortality rate attributable to 13 etiologies at global, regional, and national levels from 1990 to 2021. The findings reflect significant reductions in deaths and ASDR associated with diarrheal diseases during the COVID-19 pandemic, alongside an uneven decline in diarrhea mortality across different locations and regions. These variations may be attributed to enhancements in healthcare services, sanitation, and public health initiatives during the COVID-19 period. Notably, the increasing burden of diarrhea among adults older than 70 years presents a growing public health challenge, necessitating greater attention in light of the global aging population.

The fact that diarrhea is ubiquitous makes it a global issue, however, the burden usually falls disproportionately on people living in deprived circumstances (Chen et al., 2023). The SDI indicator, a composite metric encompassing average years of schooling, lag-distributed income per capita, and fertility rates among females younger than 25 years, has been strongly correlated with various population health outcomes and is reportedly a robust predictor of diarrheal mortality (GBD 2021 Diseases and Injuries Collaborators, 2024). The findings from this analysis reveal that regions with low-middle and low SDI exhibit the highest ASDR burdens. Specifically, countries such as South Sudan, the Republic of Chad, the Federal Republic of Somalia, the Central African Republic, and the Republic of the Niger reported ASDRs ranging from 100.00 to 166.68 per 100,000 population in 2021. These disparities highlight systemic deficiencies in health education and infrastructure, particularly in rural agricultural areas with limited access to clean water, densely populated urban informal settlements, and communities affected by crisis (Venkataramanan et al., 2020; WHO UNICEF, 2021). In addition to the resource-constrained environments, localized outbreaks of diarrhea have the potential to escalate into regional emergencies due to increasing international interactions (Fung et al., 2018). Consequently, it is essential to implement multisectoral interventions focused on the development of sanitation infrastructure and the provision of safe water. These measures are crucial for accelerating the reduction of the diarrhea burden among disproportionately affected populations, particularly undernourished children, communities lacking reliable access to safe water and adequate sanitation facilities, and individuals without access to appropriate health care in low- and middle-income countries (LMICs).

It is noteworthy that high-income North America even had the fastest rise in ASDR from 1990 to 2019, although a slight decrease was observed from 2019 to 2021. This trend may be attributed to the growing aging population in high-income countries, with the proportion of adults older than 65 years rising from 6% in 1990 to 9% in 2019. This anomaly underscores the complex interplay between demographic shifts and healthcare capacity in high-resource settings.

In 2013, WHO and United Nations International Children’s Emergency Fund (UNICEF) developed the GAPPD with the objective of eliminating preventable diarrheal deaths by 2025 (WHO UNICEF, 2013). This initiative involves the implementation of various interventions, including the provision of safe water and adequate sanitation. A specific target set for 2025 is to reduce the mortality rate from diarrhea among children younger than 5 years to fewer than 1 death per 1000 livebirths, which corresponds to a mortality rate of less than 20 deaths per 100,000 individuals. Notably, all ASDRs from diarrheal diseases have shown a decline at both the SDI and GBD region levels from 2019 to 2021. However, as of 2021, our estimates indicate a global diarrhea mortality rate of 51.72 deaths (95% UI: 38.13 to 70.54) per 100,000 children in this age group. Particularly concerning are the low SDI regions, with a mortality rate of 137.15 (95% UI: 96.81 to 190.65), and the low-middle SDI regions, with a rate of 44.63 (95% CI: 33.79 to 59.80), both of which exceed the global benchmark of 20 deaths per 100,000 among children younger than 5 years, reflecting persistent gaps in vaccine coverage, nutrition, and access to oral rehydration therapy. To decrease mortality, the action plan advocates for the promotion of a comprehensive framework of health interventions aimed at protecting, preventing, and treating diarrhea in children younger than 5 years. This includes a variety of supportive activities designed to enhance and expedite the implementation of interventions that have demonstrably reduced child mortality (Luby et al., 2018; Wagner et al., 2019; George et al., 2021; Hubbard et al., 2025). Although significant progress has been made towards achieving the established goals and coverage targets, further advancements are possible through intensified efforts in 2025. Therefore, interventions to prevent diarrheal mortality should be targeted to the unique characteristics of individual countries and regions.

In 2021, adults older than 70 years experienced a mortality rate from diarrhea that was twice as high as that of children younger than 5 years, with rates of 100.75 and 51.72 per 100,000, respectively. The total number of deaths in the older adult group was approximately 1.5 times greater than that in the younger cohort. Although the diarrhea mortality rate among adults older than 70 years has decreased by 20.7% since 2016, declining from 127.09 to 100.75 per 100,000, the absolute number of deaths showed only a marginal reduction of 5.9%, from 529,555 to 498,074, during the same period (GBD 2016 Diarrhoeal Disease Collaborators, 2018). This trend suggests that population aging has exacerbated the burden of diarrhea in this demographic. Similar to the pattern observed in children younger than 5 years, diarrhea mortality among adults older than 70 years was highest in the low SDI region, with a rate of 525.90 (95% UI: 311.47 to 864.28) deaths per 100,000. Notably, only the high SDI region exhibited a significant increase in ASDR (EAPC 5.45 [95% CI: 4.31 to 6.60]) from 1990 to 2019. Beyond the influence of various social and economic factors, biological aging itself contributes substantially to the elevated risk of diarrheal mortality. Immunosenescence, characterized by diminished mucosal and systemic immune responses, reduces resistance to enteric pathogens and vaccine effectiveness in older adults. At the same time, multiple comorbidities—including diabetes, chronic cardiovascular and renal diseases—can exacerbate dehydration and metabolic imbalance during diarrheal episodes, worsening clinical outcomes. Future research should therefore further elucidate the interactions between immunosenescence, comorbidity burden, and infection risk to inform geriatric-tailored prevention and treatment strategies.

Pathogen-specific trends provide valuable insights for setting priorities. In 2021, rotavirus was not only responsible for the highest ASDR from diarrhea but also accounted for the largest proportion of diarrheal deaths globally, particularly among children younger than 14 years. The WHO and UNICEF GAPPD also calls for increased coverage of vaccine against rotavirus, the most common cause of childhood diarrhea deaths, to reduce the disease burden substantially, as evidenced by countries that have introduced these vaccines (Burnett et al., 2020). From 1990 to 2021, rotavirus showed the largest decline (from 569100 to 131300) in global deaths, which largely attributed to vaccination efforts. Full use of the rotavirus vaccine prevented an estimated 22.0% of diarrhea deaths globally among children younger than 5 years (GBD 2017 Diarrhoeal Disease Collaborators, 2020). However, the deaths burden attributable to norovirus has been increasing over these years, with its ranking rising from the fifth to the second most common etiology in 2021. Currently, there are limited therapeutic options for norovirus, and the development of additional vaccines may be necessary. Although several vaccine candidates based on virus-like particles are being tested in clinical trials (Mattison et al., 2018; Zhang et al., 2021), the challenges to develop effective norovirus vaccines remain largely unresolved.

We have quantified the burden of different diarrheal aetiologies at different region levels, and elucidating the pathogen-specific contributions to diarrhea burden and how this varies geographically will enable interventions to be targeted. The highest burden of *Clostridium difficile* in high SDI region (ASDR 0.53 [95% UI: 0.47 to 0.61]), particularly among elderly populations, might contribute to rising diarrhea mortality because of the limited therapeutic options stemming from antibiotic resistance, frequent healthcare-associated transmission in hospital, and age-related vulnerabilities in pathogen clearance (Burke and Lamont, 2014; Smits et al., 2016; Baur et al., 2017). *Cryptosporidium* was notably different from *Clostridium difficile* in that a highest burden was observed in the low SDI region, and almost exclusively in Sub-Saharan Africa. Therapeutic options for *Cryptosporidium*are severely limited (Lu et al., 2025), and no vaccine candidates have advanced to clinical trials, revealing a substantial gap in treatment and prevention. All these findings implies that advancements in national infrastructure and healthcare financing associated with economic growth enable governments to build and maintain integrative surveillance and treatment programs, thereby reducing fragmentation in public health responses. For example, China initiated comprehensive national health reforms in the early 1990s targeting children younger than 5 years mortality reduction (Guo et al., 2015). A cornerstone of these efforts was the National Diarrheal Disease Control Program, which implemented a multi-tiered strategy encompassing nationwide surveillance systems, community-based health education campaigns, healthcare worker training initiatives, and equitable healthcare access expansion. This program adopted a geographically inclusive approach, covering all provincial-level administrative units while prioritizing resource allocation to rural regions where diarrheal disease burden was most pronounced.

Nevertheless, the ASDRs for multiple pathogens increased in East Asia during the pandemic, contrasting the global decline. This anomaly can be largely attributed to the region’s distinctive public health response. Firstly, the exceptionally stringent and prolonged NPIs, while effective against COVID-19, likely disrupted healthcare access, leading to delayed diagnosis and treatment of severe diarrhea. Secondly, the widespread empirical use of antibiotics for respiratory infection prophylaxis may have promoted dysbiosis and antimicrobial resistance, exacerbating outcomes for bacterial enteropathogens. Critically, these interventions also created a substantial ‘immunity gap’—a phenomenon highlighted in recent scientific discourse (Rubin, 2024). The prolonged suppression of enteric pathogen circulation left a larger population susceptible, setting the stage for more intense resurgences once restrictions eased. Thus, the observed increase in East Asia likely reflects a complex interplay of healthcare disruption, altered pharmaceutical practices, and a temporary, population-wide immunity gap.

Although global mortality from diarrheal diseases have decreased, the progress achieved remains tenuous and unevenly distributed. Factors such as aging populations, antimicrobial resistance, and climate-related disruptions pose significant risks of reversing these advancements. Future strategies should incorporate targeted vaccination initiatives, robust health system enhancements, and adaptive policies that address both the socioeconomic determinants and the challenges posed by emerging pathogens. It is imperative to prioritize vulnerable groups, especially children in low SDI region and elderly populations worldwide, to ensure sustainable reductions in the burden of diarrheal diseases.

This study has several limitations. First, the pathogen-specific estimates, derived from a model-based counterfactual approach, are subject to potential ecological fallacy, as population-level attributions may not reflect individual-level causes of death. This method also critically depends on the quality of input data and required extrapolating odds ratios from pediatric studies (GEMS) to all age groups—an assumption that requires further validation. Second, data limitations, particularly in low-surveillance settings, necessitated reliance on verbal autopsy and spatial modelling. While these methods compensate for data gaps, they widen uncertainty intervals and risk underreporting or misclassifying pathogens that are difficult to diagnose or under-surveilled (e.g., *Clostridium difficile* in LMICs). Finally, regarding the accelerated decline in ASDR during 2019-2021, our analysis could not fully disentangle the impact of public health interventions from potential under-ascertainment caused by healthcare disruptions and reduced healthcare-seeking. Thus, the observed trend may overstate the true mortality reduction.

## Conclusion

Our analysis from 1990 to 2021 reveals a transformative period in the global diarrheal disease landscape, marked by an accelerated decline in mortality during the COVID-19 pandemic, a shifting burden toward adults aged ≥70 years, and the growing prominence of pathogens such as norovirus (**Supplementary Figure S4**). To consolidate these gains and address persistent inequities, public health strategies must evolve through coordinated, multi-sectoral approaches. The demographic shift identifying older adults as the highest-risk group calls for greater investment in SDI-adapted WASH programs tailored to aging populations. Meanwhile, the persistent dominance of rotavirus underscores the need to sustain and expand vaccine coverage, while the rise of norovirus and the substantial burden of *Cryptosporidium* in low-resource settings highlight critical gaps in the vaccine development pipeline, necessitating intensified research and investment. Achieving equitable and sustainable control will therefore require reinforced vaccination, SDI-adapted WASH interventions, and strengthened global pathogen surveillance systems.

## Data Availability

The original contributions presented in the study are included in the article/**Supplementary Material**. Further inquiries can be directed to the corresponding author.

## Supplementary material

Supplementary Figure 1 Ranked aetiologies by age-standardized mortality rate of diarrheal diseases across all ages by country or territory. NTS, Non-typhoidal Salmonella; Adeno, Adenovirus; Aero, Aeromonas; Campy, Campylobacter; C diff, Clostridium difficile; Crypto, Cryptosporidium; EPEC, enteropathogenic E. coli; ETEC, enterotoxigenic E. coli; Noro, Norovirus; Rota, Rotavirus.

Supplementary Figure 2 EAPCs in age-standardized mortality rate in 1990-2019 (left) and 2019-2021 (right) by country.

Supplementary Figure 3 Association between the SDI and diarrheal diseases age-standardized mortality rate across all ages by region, separated by pathogen, 1990-2021. SDI, Socio-demographic Index. GBD, Global Burden of Disease. The solid lines are estimated by a least squares cubic spline regression.

Supplementary Figure 4 A graphical summary of key findings in the global burden of diarrheal diseases, 1990-2021.
