# Supplementary material for: The GBD 2021 perspective: COVID-19’s impact on diarrheal mortality and etiological trends, 1990–2021
Source: Front Cell Infect Microbiol. 2025 Nov 19;15:1668444. doi: 10.3389/fcimb.2025.1668444 (PMC12672554; doi:10.3389/fcimb.2025.1668444)
Supplement: Supplementary Figure 1 — Ranked aetiologies by age-standardized mortality rate of diarrheal diseases across all ages by country or territory. NTS, Non-typhoidal Salmonella; Adeno, Adenovirus; Aero, Aeromonas; Campy, Campylobacter; C diff, Clostridium difficile; Crypto, Cryptosporidium; EPEC, enteropathogenic E. coli; ETEC, enterotoxigenic E. coli; Noro, Norovirus; Rota, Rotavirus. [file DataSheet1.pdf]

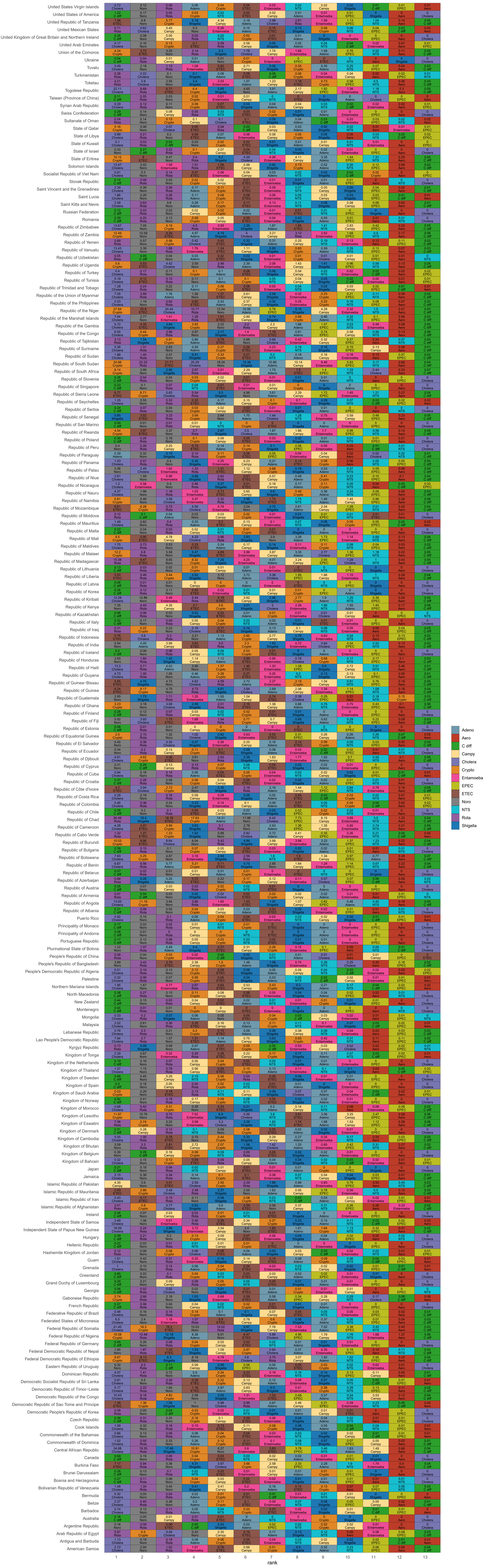

Adeno
Aero
C diff
Campy
Cholera
Crypto
Entamoeba
EPEC
ETEC
Noro
NTS
Rota
Shigella
rank
1 2 3 4 5 6 7 8 9 10 11 12 13
