# Supplementary figures and images for: The GBD 2021 perspective: COVID-19’s impact on diarrheal mortality and etiological trends, 1990–2021
Source: Front Cell Infect Microbiol. 2025 Nov 19;15:1668444. doi: 10.3389/fcimb.2025.1668444 (PMC12672554; doi:10.3389/fcimb.2025.1668444)

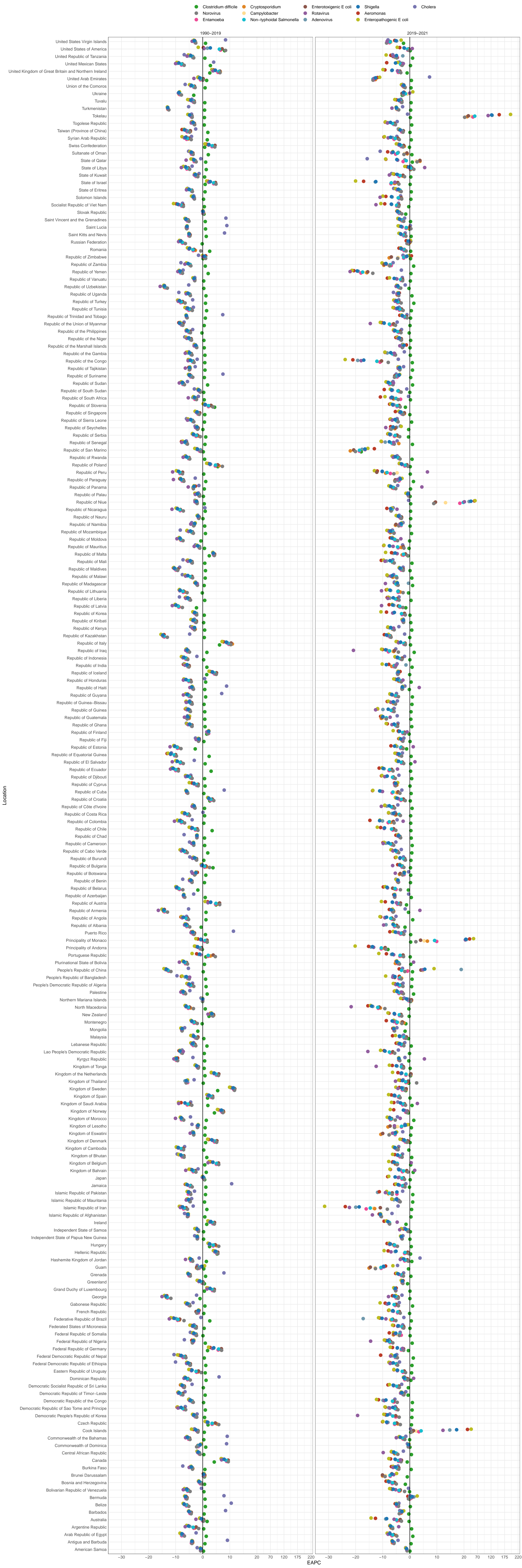

Supplement: Supplementary Figure 2 — EAPCs in age-standardized mortality rate in 1990-2019 (left) and 2019-2021 (right) by country. [file DataSheet2.pdf]

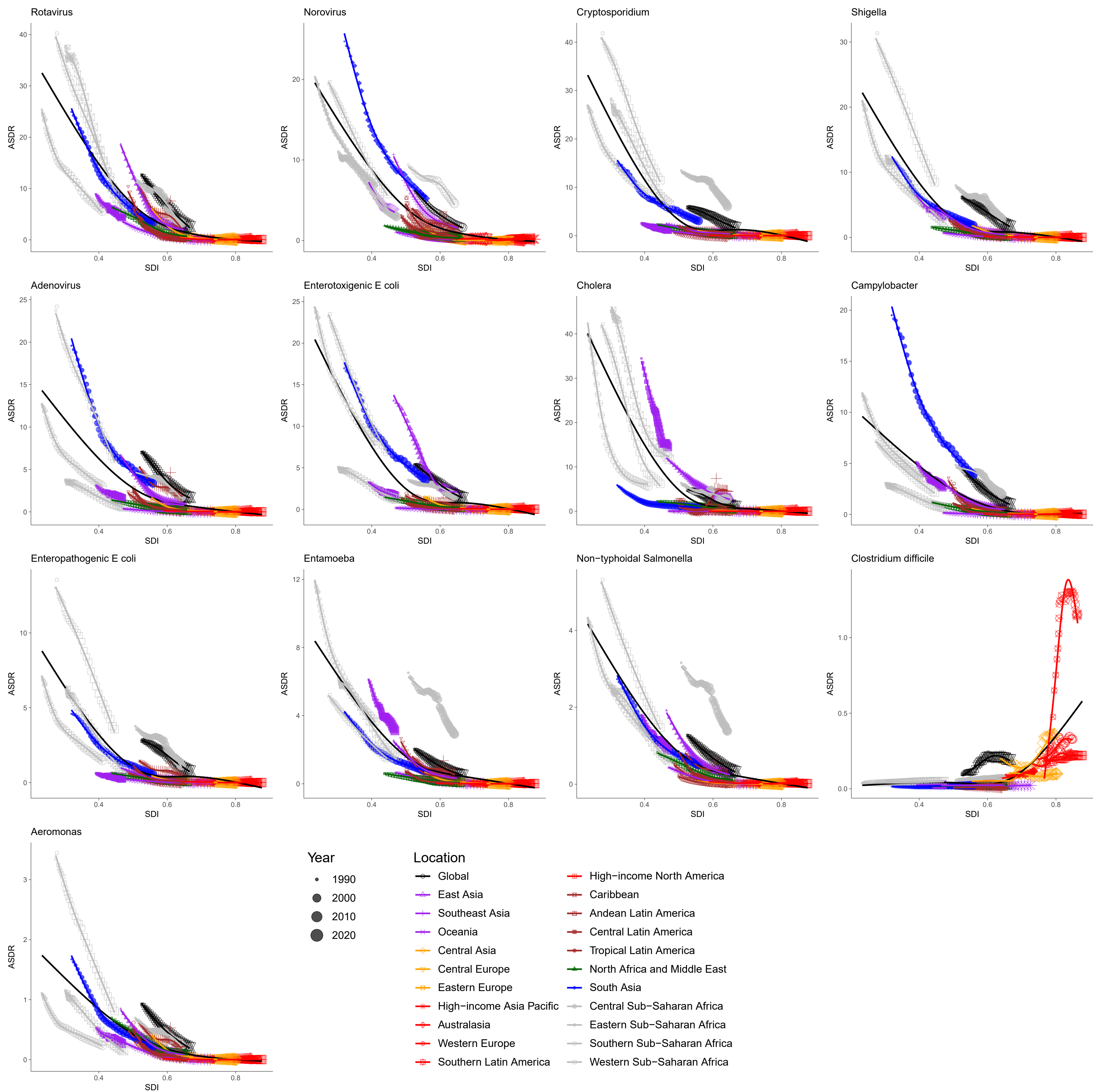

Supplement: Supplementary Figure 3 — Association between the SDI and diarrheal diseases age-standardized mortality rate across all ages by region, separated by pathogen, 1990-2021. SDI, Socio-demographic Index. GBD, Global Burden of Disease. The solid lines are estimated by a least squares cubic spline regression. [file DataSheet3.pdf]
