# Supplementary material for: The GBD 2021 perspective: COVID-19’s impact on diarrheal mortality and etiological trends, 1990–2021
Source: Front Cell Infect Microbiol. 2025 Nov 19;15:1668444. doi: 10.3389/fcimb.2025.1668444 (PMC12672554; doi:10.3389/fcimb.2025.1668444)
Supplement: Supplementary Figure 4 — A graphical summary of key findings in the global burden of diarrheal diseases, 1990-2021. [file DataSheet4.pdf]

- Global diarrheal deaths declined faster during COVID-19.
- Burden concentrated in low-SDI regions, highlighting stark inequity.
- Adults >70 surpassed under-5 as highest-risk group.

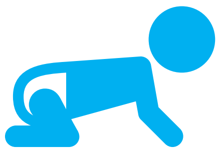

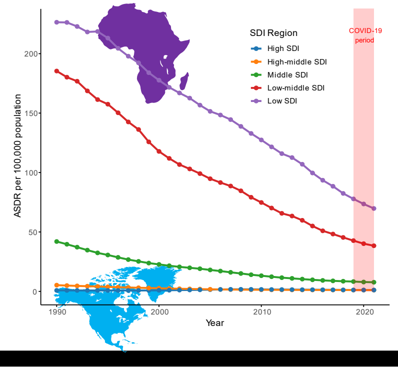

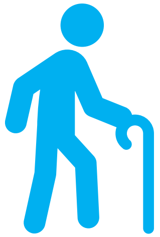

1990 — 2021

- Rotavirus led deaths, norovirus rose to second.

1990	2019	2021
1. Rotavirus	1. Rotavirus	1. Rotavirus
2. Adenovirus	2. Shigella	2. Norovirus
3. *Shigella*	3. *Cryptosporidium*	3. *Cryptosporidium*

- Public Health Implication

Need for SDI-adapted WASH programs, particularly for aging populations in low-SDI regions

WASH WATER SANITATION AND HYGIENE

Critical gaps in vaccine development for norovirus and *Cryptosporidium*, necessitating research and investment
